# Harnessing strontium in regenerative dentistry: progress and perspectives

**DOI:** 10.3389/fbioe.2025.1724610

**Published:** 2025-11-21

**Authors:** Wenjing Zhang, Lingfei Wei

**Affiliations:** Department of Oral Implantology, Yantai Stomatological Hospital, Binzhou Medical University, Yantai, China

**Keywords:** strontium, strontium-doped biomaterials, oral tissue repair, cell regulation, clinical application

## Abstract

Strontium (Sr), an alkaline earth element characterized by its unique bioactivity, has garnered significant interest in the field of oral regenerative medicine. This interest is primarily due to its inherent presence in bone tissues, particularly in growth plates and trabeculae, and its ability to influence bone metabolism through the calcium-sensing receptor (CaSR). Various signaling pathways, including MAPK/ERK1/2 and Wnt/NFATc, are involved in the mechanisms behind these effects. Strontium-doped biomaterials progressively release Sr^2+^ ions, which impart a variety of biological effects, among these are the stimulation of osteoblast proliferation and differentiation, and the exhibition of antibacterial and anti-inflammatory characteristics. Moreover, these ions are involved in regulating the deposition of the extracellular matrix. Collectively, these characteristics underscore the potential utility of strontium-doped biomaterials for the regeneration of soft and hard tissues, exemplified by applications in alveolar bone reconstruction and the osseointegration of dental implants. Despite these advantages, numerous challenges persist, particularly in relation to degradation kinetics, long-term *in vivo* stability, and translational applications. This review offers a comprehensive examination of the biological roles of strontium, recent advancements in its integration into biomaterials, and the underlying molecular mechanisms. Additionally, it addresses current limitations and proposes potential strategies for optimization, thus offering a theoretical basis for the future creation of novel oral regenerative materials.

## Graphical Abstract

**Figure:**
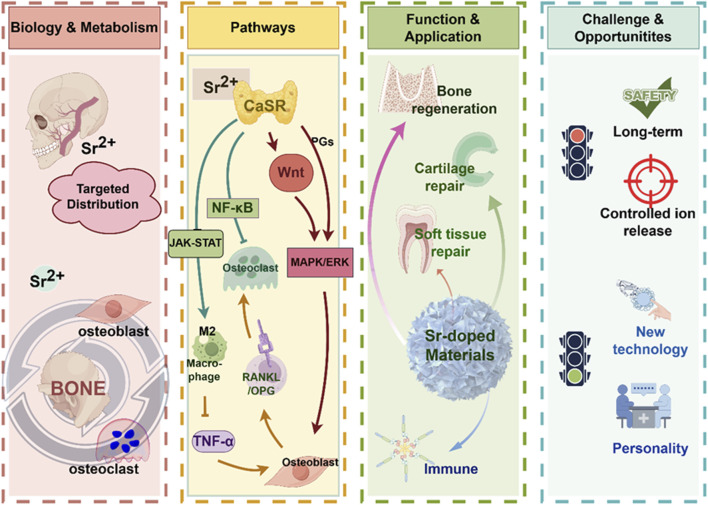

## 1 Introduction

The rising incidence of oral diseases among aging populations has positioned oral regenerative medicine as a pivotal area of focus within biomedical research. Achieving effective regeneration of oral hard and soft tissues continues to represent a fundamental challenge for both clinical dentistry and the field of biomaterials science, Contemporary therapeutic objectives encompass the consistent reconstruction of alveolar bone post-tooth extraction, dependable osseointegration of dental implants, restoration of periodontal and pulpal tissues, and the functional rehabilitation of the temporomandibular joint. While traditional grafts and biomaterials have enhanced clinical outcomes, their restricted bioactivity and inadequate capacity to modulate the tissue microenvironment underscore the necessity for exploring more sophisticated regenerative approaches.

Strontium (Sr), an alkaline earth element with chemical properties akin to calcium, has gained recognition as a promising candidate in this context. Strontium is primarily introduced into the body through dietary consumption and is distributed across various tissues, exhibiting a pronounced affinity for areas of active bone remodeling (Ru et al., 2024). Notably, elevated concentrations are consistently observed in trabecular regions, such as the mandible and craniofacial bones, in contrast to cortical sites like the femur. The selective accumulation of strontium has obtained significant attention regarding its biological role in skeletal metabolism (Panahifar et al., 2019). Researches have shown that Strontium ions (Sr^2+^) trigger calcium-sensing receptors (CaSR) on osteoblasts and osteocytes, activating signaling pathways like mitogen-activated protein kinase (MAPK) pathways and Wnt/NFATc. These pathways promote the growth and specialization of osteoblasts, and simultaneously suppresses the activity of osteoclast. It ensures a balanced control of bone regeneration and absorption. The dual action differentiates strontium from numerous other alkaline earth ions and provides compelling evidence for its incorporation into regenerative biomaterials.

In addition to the effect on bone metabolism, strontium exhibits other biological functions of the more comprehensive process of oral tissue repair, including the anti-inflammatory effect, antibacterial effect, and ECM deposition modulation. These properties may make strontium more applicable for soft tissue repair in addition to bone regeneration because infection control and immune modulation are also important for soft tissue repair. In other words, the biological multifunctional properties of strontium suggest that it may not only be able to participate in the reconstruction of the structure but also be able to create a more favorable microenvironment for tissue repair.

**FIGURE 1:**
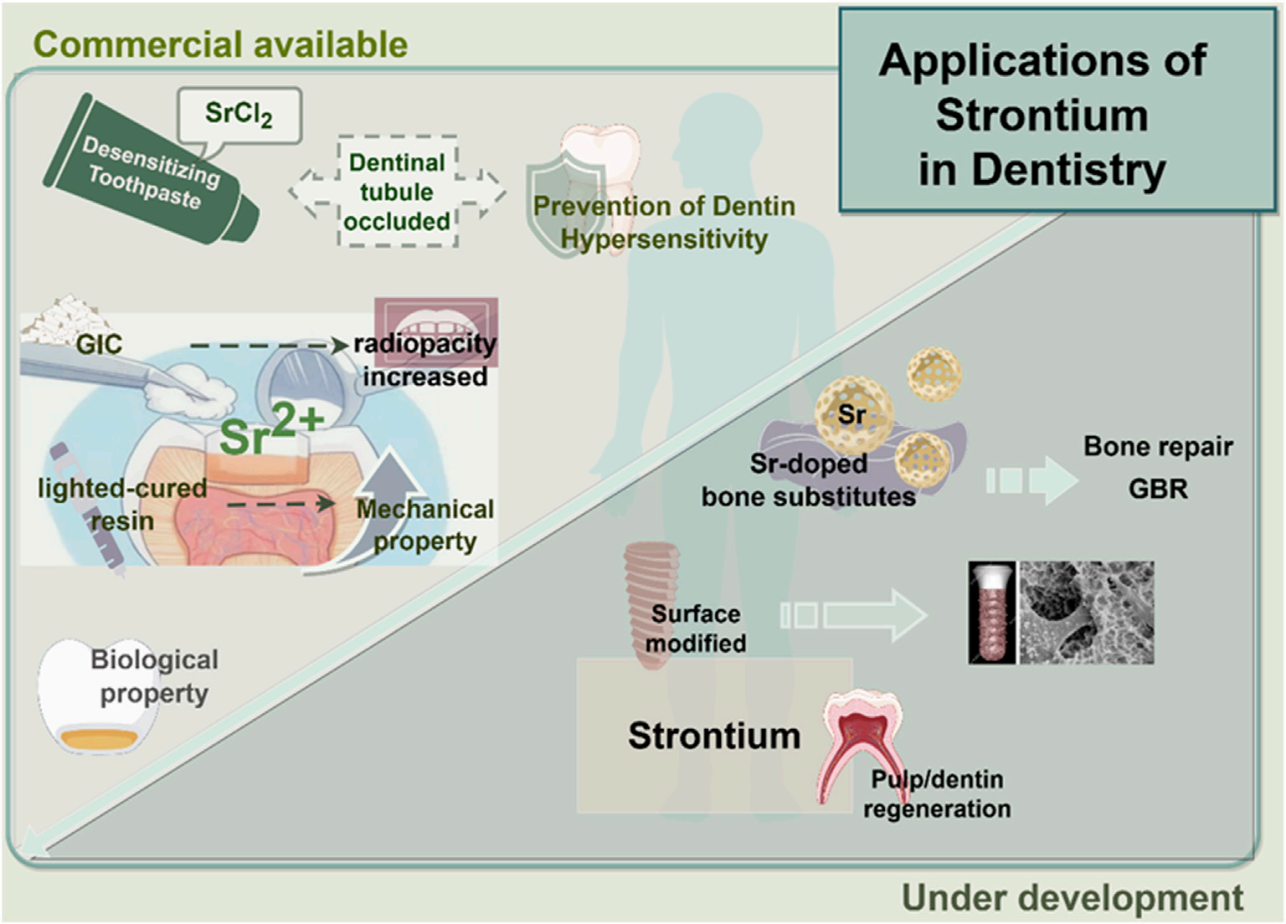
Application of strontium in dentistry. This encompasses the integrated use of strontium with dental or healthcare materials in clinical practice, in addition to tissue repair materials that are presently in the experimental phase.

Based on the above studies, strontium-doped biomaterials with different compositions have been prepared and studied. It has been reported that the crystal structure and chemical characteristics of these biomaterials have been changed by doping Sr^2+^. The bioactivity of these biomaterials has been improved (Huang et al., 2022). In addition, both *in vivo* and *in vitro* experiments have shown that strontium-doped biomaterials could enhance the bone formation and accelerate the mineralization process of the newly formed bone with better quality of regenerated bone tissue (Bosch-Rué et al., 2020). Moreover, the release of Sr^2+^ could present antibacterial advantages so that the risk of infection at the implant site could be reduced or when performing bone grafting procedures. In addition, some reports (Li P. et al., 2024; Tie et al., 2020) have explored the synergistic effect when doping strontium with other ions with biological activities (Figure 1). However, several challenges persist in the clinical translation of strontium-doped materials. One significant technical challenge is the control of degradation kinetics, which means a consistent release of ions without compromising the stability of the scaffold. Moreover, the long-term safety and systemic effects of these materials, as well as the consistency of results across different defect types and experimental sites should be clarified by further researches. Furthermore, the majority of evidence currently available is derived from preclinical models, so the rigorous clinical trials are needed to establish efficacy in human patients. These barriers highlight the importance to integrate mechanistic insights with translational research. It will facilitate the transition of strontium-doped biomaterials from the laboratory to clinical practice.

This review seeks to synthesize existing findings on the biological characteristics and metabolic roles of strontium, assess recent advancements in the advancement of strontium-doped biomaterials, and explores their potential function in oral tissue regeneration. Unlike previous reviews on strontium-based biomaterials, which have primarily concentrated on their critical role in the repair and regeneration of various tissues throughout the human body, this article emphasizes a more in-depth investigation into the reparative potential of strontium-doped biomaterials specifically within the oral context. By elucidating the molecular mechanisms underlying the bioactivity of strontium, analyzing existing challenges, and proposing strategies to overcome them, this targeted exploration aims to more effectively facilitate the translation of laboratory findings into clinical practice. By incorporating biological insights with material science perspectives, offers a comprehensive framework for advancing based on strontium innovations in the field of regenerative dentistry.

## 2 Biological characteristics of strontium ions

**FIGURE 2:**
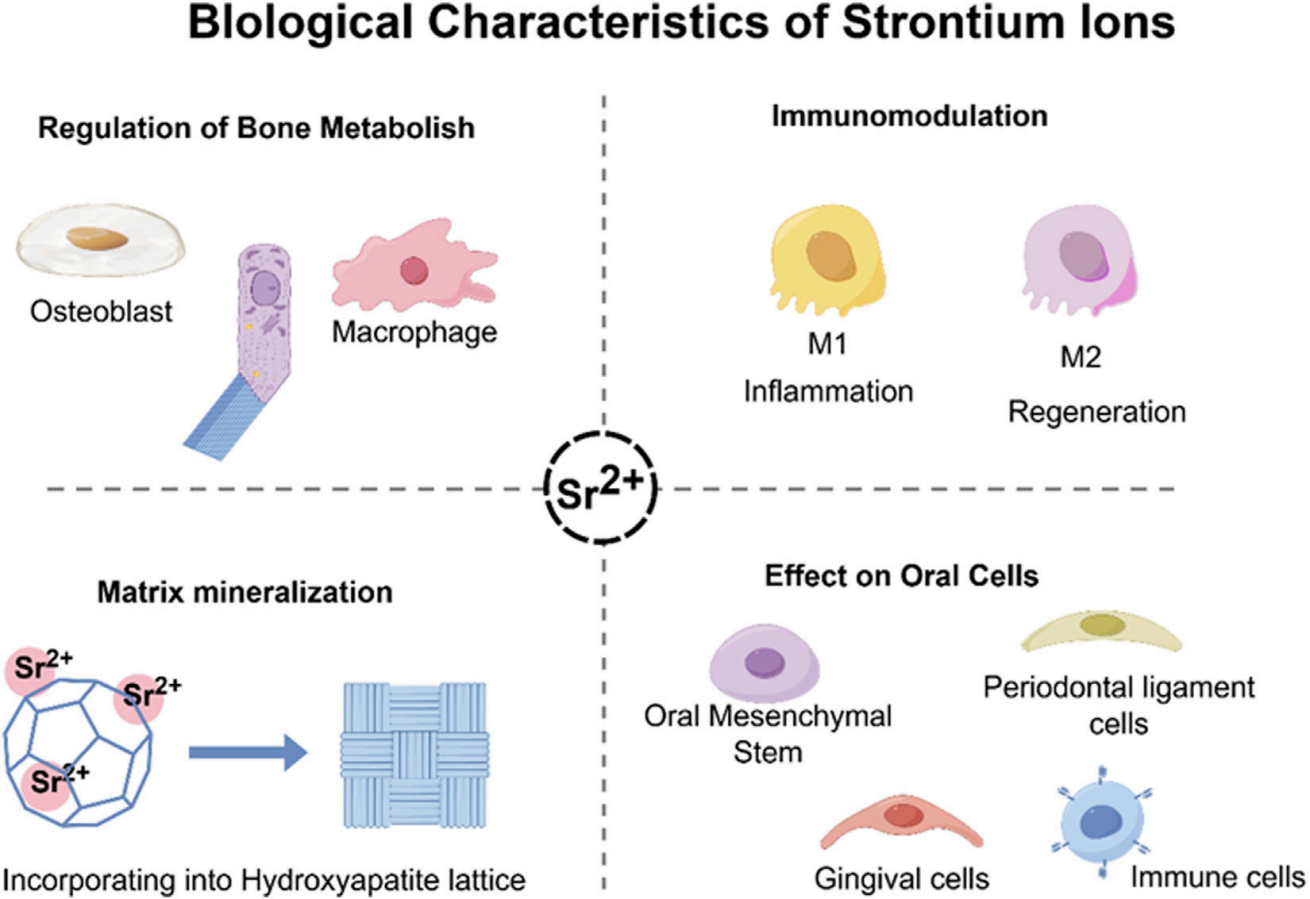
Biological characteristics of strontium ions. It can promote osteogenesis and mineralization by regulating the balance of intercellular interactions *in vivo*, while modulating the *in vivo* osteoimmune microenvironment.

It has garnered significant interest within the field of regenerative medicine due to the unique biological properties of Sr^2+^, especially its concerning applications in bone and oral tissue repair (Wang et al., 2019) (Figure 2). Clinical data derived from the administration of strontium ranelate has showed that Sr^2+^ can reduce the incidence of both vertebral and non-vertebral fractures for its bifunctional effects of promoting osteogenesis and inhibiting osteoclastic activity *in vivo*. These clinical observations lay the groundwork for further investigation into the cellular and molecular mechanisms by which Sr^2+^ facilitates tissue regeneration.

### 2.1 Regulation of strontium ions for bone metabolism

Sr^2+^ could improve the bone metabolism by stimulating bone formation and inhibiting osteoclast bone resorption (Kong et al., 2025). Firstly, Sr^2+^ could upregulate the MAPK/ERK signaling pathways mechanistically activated by CaSR to promote osteoblast growth, maturation, and mineral deposition. Upregulation of osteogenic genes such as *Runx2, Osterix, ALP, OCN, Col-I* (Zhang et al., 2025; Chen K. et al., 2025) as well as the expression of alkaline phosphatase and collagen to form the bone matrix. Secondly, Sr^2+^ can prolong the osteoblast life span by inhibiting osteoblast apoptosis. In addition, regarding to, Sr^2+^ can play an anti-resorptive role by modulating the RANKL/OPG signaling pathway to inhibit osteoclast genesis. Sr^2+^ can enhance the osteoprotegerin (OPG) expression in osteoblasts and bone stromal cells while reducing the RANKL expression. This will lead to the suppression of osteoclast differentiation and activity. Finally, Sr^2+^ can attenuate the nuclear factor kappa-light-chain-enhancer of activated B cells (NF-κB) signaling and further suppress osteoclast mediated bone resorption. In short, these processes will create a favorable microenvironment which can accelerate the bone regeneration.

### 2.2 Immunomodulatory functions of strontium ions

Recently, Sr^2+^ essential function in modulating the bone immune microenvironment was also reported, especially on the effect of Sr^2+^ on macrophage polarization was widely concerned (Wu et al., 2025). Sr^2+^ could decrease the expression of tumor necrosis factor-alpha (*TNF-α*), interleukin (*IL)-1β*, *IL-6* and further inhibited the M1 macrophage phenotype (Shefa and Kim, 2025). On the contrary, Sr^2+^ could enhance the expression of *IL-10, TGF-β, VEGF*, morphogenetic protein-2 (BMP-2) and promote the transformation of M2 macrophage phenotype. This macrophage polarization process can also facilitate the debris clearance, angiogenesis and osteogenic factor release. This phenotype can build an anti-inflammatory environment (Zhu et al., 2016; Wei et al., 2018). In short, Sr^2+^ can effectively regulate the intensity and duration of the local inflammatory environment and build an optimal environment for tissue repair.

### 2.3 Effects of strontium ions on matrix mineralization

Because of their chemical similarity to Ca^2+^, Sr^2+^ can enter the hydroxyapatite matrix of the bone mineral matrix (Baheiraei et al., 2021), and compete with calcium in calcium-binding proteins such as calmodulin and CaSR, which affect subsequent signaling pathways (Cheshmedzhieva et al., 2021). Therefore, it is possible that Sr2^+^ can promote biocompatibility and osseointegration of the implant (Kruppke et al., 2019). Compared to Ca^2+^, Sr^2+^ induces longer lasting phosphorylation of extracellular signal-regulated kinase (ERK) so that it has a better effect on matrix mineralization (Querido and Farina, 2013; Yamaguchi and Weitzmann, 2012).

## 3 Cellular regulatory mechanisms of strontium ions in oral tissue repair

Sr^2+^ is involved in the repair of oral tissues by regulating different signaling pathways, cell differentiation and tissue repair.

### 3.1 Oral mesenchymal stem cells

Relevant researches revealed that Sr^2+^ can enhance the pluripotent differentiation of mesenchymal stem cells (MSCs) via multiple pathways.

**FIGURE 3:**
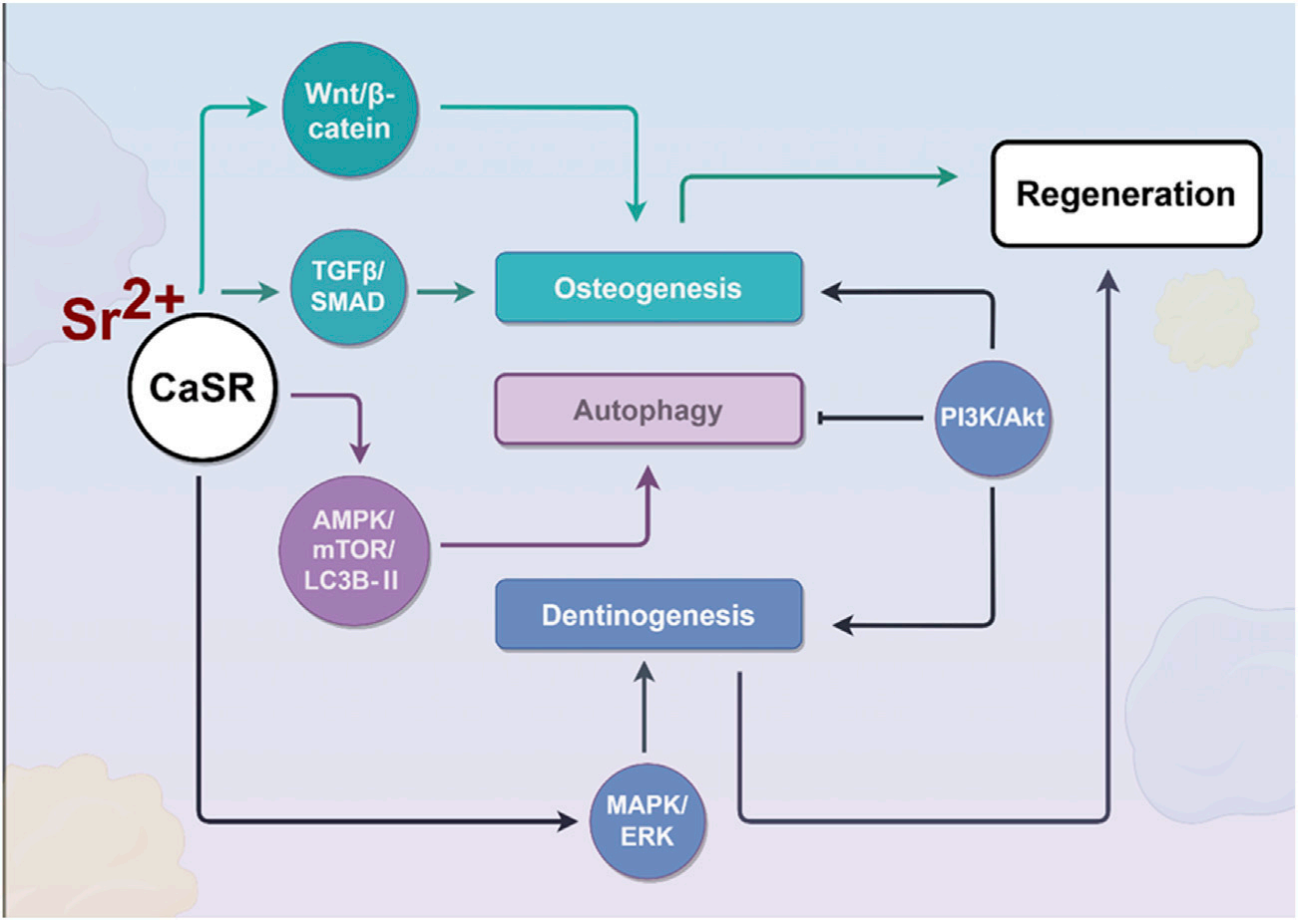
Effects of strontium ions on the process of bone regeneration. During osteogenesis, Sr^2+^ activates the CaSR, which subsequently triggers downstream signaling cascades, including the Wnt/β-catenin, TGFβ/SMAD, and MAPK/ERK pathways, to promote osteogenic differentiation of cells. Furthermore, by activating the AMPK/mTOR/LC3B-II signaling pathway, Sr^2+^ enhances cellular autophagy, thereby effectively delaying cellular senescence.

During osteogenesis, Sr^2+^ activates the Wnt/β-catenin signaling pathway, promoting the proliferation and osteogenic differentiation potential of MSCs, promoting their transformation into osteoblasts (Wei et al., 2018; Sun et al., 2025; Bizelli-Silveira et al., 2018). In addition, Sr^2+^ interacts with the CaSR to affect cellular chemical components, promoting osteoblast differentiation and mineralization. As shown in Figure 3, strontium phosphate activates the CaSR promotes their proliferation and mineralization by phosphorylating ERK in osteoblasts (Takaoka et al., 2010). Sr^2+^ promotes the formation of new bones in living bodies by promoting the proliferation and differentiation of primary bovine chondrocytes into bones through the TGFβ/SMAD signaling pathway (Yang et al., 2011; Tsai et al., 2018). From the above research, we have a theoretical basis to apply Sr^2+^ in the regeneration and repair of oral and craniofacial defects.

Sr^2+^ affects the transformation of MSCs into dental pulp stem cells (DPSCs), which in turn enhance the subsequent osteogenic differentiation (Tang et al., 2025; Basheer et al., 2021). Research on DPSCs have shown that Sr^2+^ greatly enhances the adhesion, growth, and differentiation of cells. Sr^2+^ also plays an important role in promoting dental pulp stem cells to differentiate into odontoblasts.

They enhance the proliferation and differentiation of these cells by several means (Miyano et al., 2023). Firstly, this is realized by activating CaSR, and the following MAPK/ERK signaling pathway, as shown in Figure 3, similar to the process of osteoblast differentiation (Huang et al., 2016). This can be realized by upregulating the expression of the *Runx2, DSPP,* and *DMP1* genes (Zamir Nasta et al., 2024). Namely, strontium, as a divalent cation, exhibits chemical similarities to Ca^2+^ and can mimic calcium in binding to the CaSR, thereby initiating downstream signaling pathways, including the Wnt/β-catenin and MAPK pathways (Conigrave and Ward, 2013; Ishitani et al., 2003). The Wnt/β-catenin pathway is essential for regulating cell proliferation, differentiation, and tissue regeneration. The binding of Wnt ligands to cell surface receptors inhibits the degradation of cytoplasmic β-catenin, resulting in its accumulation and subsequent translocation to the nucleus (Wang H. et al., 2024). Within the nucleus, β-catenin activates the transcription of target genes, thereby facilitating cell proliferation and differentiation. The MAPK pathway includes classical subfamilies such as ERK, JNK, and p38. Research has shown that the Wnt-5a-induced Wnt/Ca^2+^ pathway can counteract Wnt/β-catenin signaling by activating the TAK1-NLK MAPK cascade. Overall, strontium influences cell proliferation, differentiation, matrix synthesis, and calcium homeostasis in oral tissue repair by modulating the Wnt/β-catenin, MAPK, and CaSR-mediated signaling pathways.

In addition, Sr^2+^ enhances the growth and odontogenic/osteogenic differentiation of dental pulp cells by triggering the PI3K/Akt signaling pathway (Bakhit et al., 2018).

Sr^2+^ plays an important role in postponing cellular aging by enhancing autophagy. They can enhance autophagy in fibroblast-like synoviocytes through the AMPK/mTOR/LC3B-II signaling pathway, then postponing cellular aging (Liao et al., 2023). It is likely that Sr^2+^ will also work in a similar way in dental stem cells, postponing cellular aging and promoting dental stem cell regeneration and repair.

### 3.2 Osteoclast

Sr^2+^ enhance the OPG expression level via up-regulating LRP6/β-catenin pathway which can inhibit the signaling pathway of NF-κB and affect the activity of osteoclast; Through above two ways, the process of RANKL-induced osteoclastogenesis is inhibited and the bone resorption will be decreased (Sun et al., 2019; Huang et al., 2020). Sr^2+^ enhance the OPG expression level via up-regulating LRP6/β-catenin pathway which can inhibit the signaling pathway of NF-κB; Besides, Sr^2+^ can affect the activity of osteoclast via impeding the signaling pathway of NF-κB. The bone resorption will be decreased (Aimaiti et al., 2017).

### 3.3 Periodontal ligament cells

The effects of Sr^2+^ on periodontal ligament cells have also been studied in researches (Er et al., 2008). The results demonstrated that the Sr^2+^ concentration has a promoting effect on the growth of periodontal ligament cells, and could promote the process of bone formation, such as enhancing the activities of alkaline phosphatase and promoting mineralized nodule formation (Bizelli-Silveira et al., 2018). Sr^2+^ is expected to be used in periodontal bone tissue engineering.

### 3.4 Gingival fibroblast

Preliminary studies reveal that Sr^2+^ has a positive effect on human gingival fibroblasts (Alsharif et al., 2023; Bakitian et al., 2024). *In vitro* experiments revealed that the addition of strontium citrate (0.5 mM–1.0 mM) increases the activity of human gingival cells and decreases cell death (Fernandes et al., 2019). Based on research using scanning electron microscopy, the inclusion of strontium can enhance the attachment of gingival fibroblasts to the healing abutment surface and thus provides a basis for the prevention and treatment of peri-implantitis by enhancing the soft-biomembrane barriers around the implant.

### 3.5 Immune cells

Sr^2+^ have a significant impact on oral immune responses, especially in pathological conditions such as periodontitis (Matvieienko et al., 2022; Zhang et al., 2014) (Figure 5). Specifically, Sr^2+^ have been demonstrated to: (1) Inhibit the production of lipopolysaccharide (LPS)-induced pro-inflammatory cytokines in human periodontal ligament cells (hPDLCs); (2) Restore the expression of early osteogenic genes, which are commonly suppressed by inflammatory processes; (3) Promote bone regeneration during the initial phases of osteogenic differentiation, although its impact on mineralization in the later stages may be variable.

**FIGURE 4:**
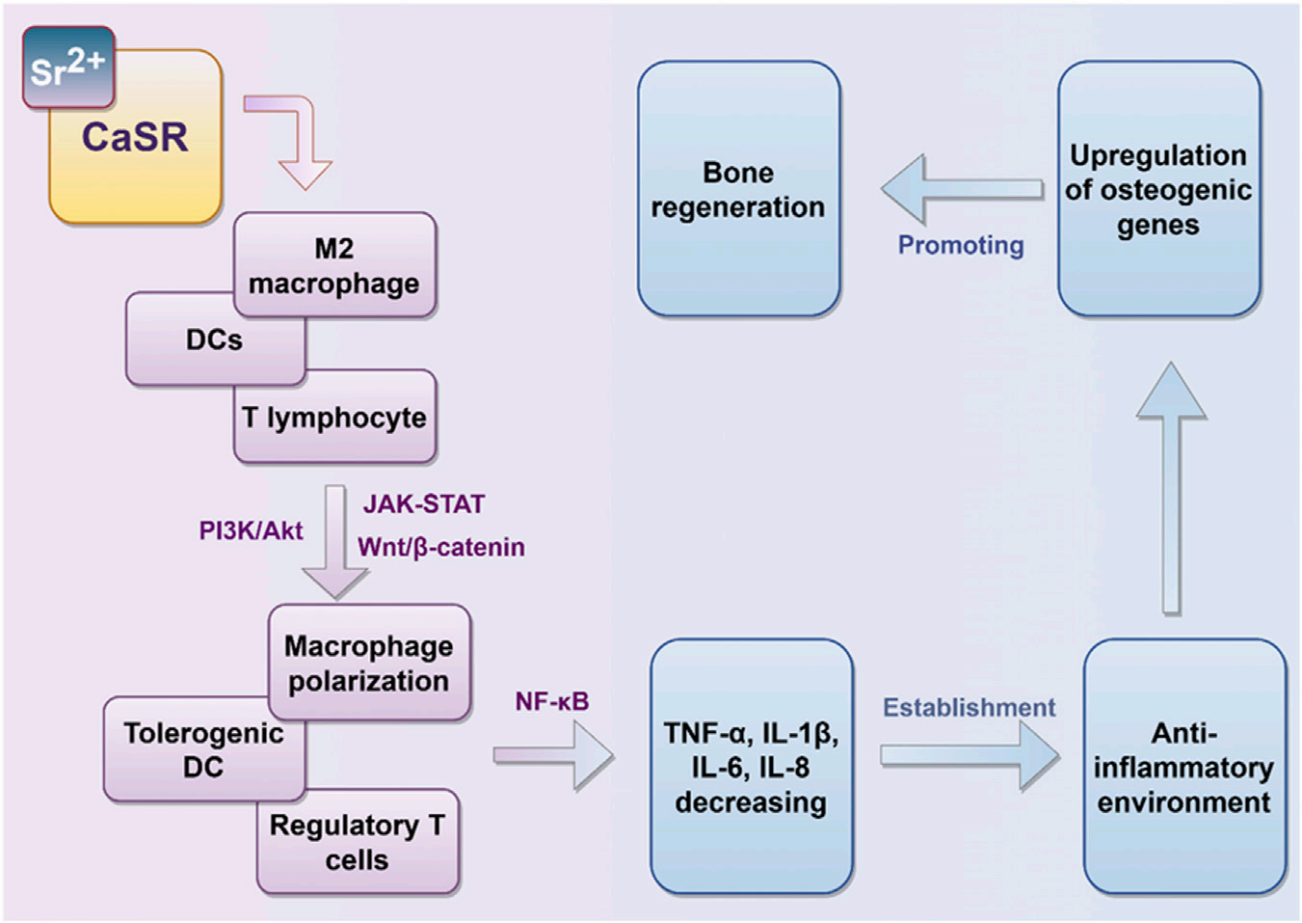
Immunomodulatory effects of strontium ions. Sr^2+^ modulates the polarization state of immune cells *in vivo*, thereby inhibiting the secretion of pro-inflammatory mediators, fostering an anti-inflammatory microenvironment, and facilitating conditions conducive to bone regeneration.

Strontium predominantly activates the CaSR on macrophages, dendritic cells, and T cells. This activation modulates the phenotypic transition of these immune cells through the activation of the PI3K, Wnt/β-catenin, and JAK-STAT signaling pathways. Consequently, it inhibits the release of pro-inflammatory factors by engaging the NF-κB signaling pathway, thereby fostering an anti-inflammatory milieu (Figure 4). Sr^2+^ are able to participate in pro-inflammatory milieu establishment in oral cavity. Particularly, effect on regulating the polarization phenotype of macrophages is crucially important. In this study, we explored the way of Sr^2+^ influence the growth, inflammation control and bone differentiation of hPDLCs in diseased states.

The hPDLCs demonstrated significantly enhanced growth ability when treated with different concentrations of Sr^2+^ (ranging from 0.02 to 2.5 mmol/L). Sr^2+^ could significantly suppress the expression of pro-inflammatory cytokines, including *TNF-α*, *IL-1β*, *IL-6* and *IL-8* induced by lipopolysaccharide (LPS) stimulation. Our results demonstrated the importance of Sr^2+^ in inflammatory reaction (Wei et al., 2018). Interestingly, Sr^2+^ were able to rescue the early osteogenic differentiation genes suppressed by LPS, but exhibited inhibitory effect on later mineralization process.

## 4 Advances in research on strontium-doped biomaterials for oral tissue repair

**FIGURE 5:**
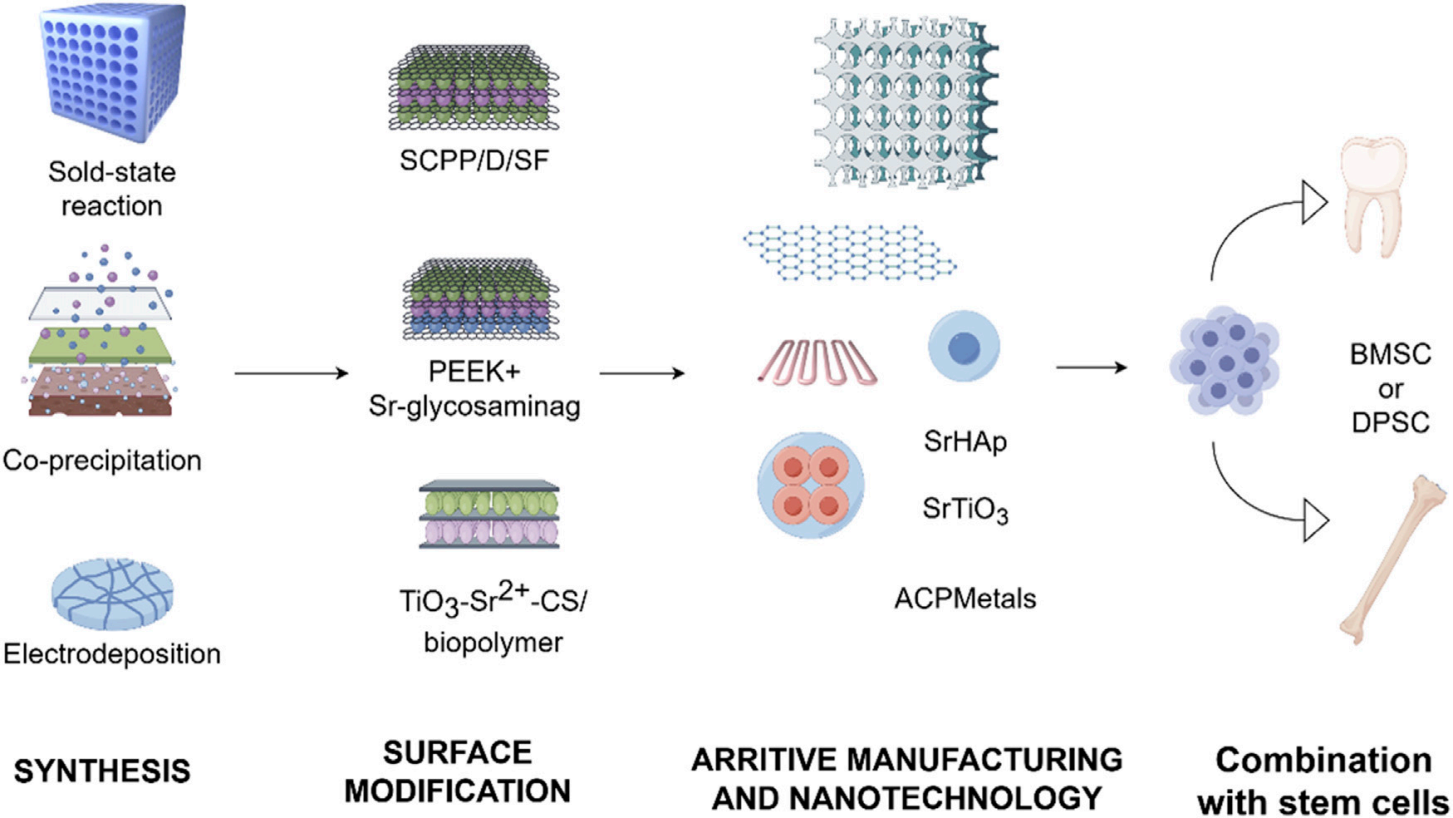
Progress in the development of strontium-doped materials. The development of strontium-doped biomaterials has advanced from initial physicochemical integration to the investigation of surface functionalization. This progression has increasingly embraced additive manufacturing, the application of nanotechnology, and more comprehensive integration with stem cell technologies.

As discussed in earlier chapters, strontium has attracted significant interest in oral regenerative medicine because of its bone-forming and immune-modulating characteristics. Doping strontium into biomaterials has been demonstrated to greatly improve bone regeneration and integration, making strontium-doped biomaterials promising options for oral tissue repair. This section delves into recent advancements concerning the preparation, surface functionalization, additive manufacturing, nanotechnology applications, and stem cell integration of strontium-doped biomaterials (Figure 5).

### 4.1 Preparation and functionalization of strontium-doped biomaterials

#### 4.1.1 Synthesis methods

Traditional techniques for synthesizing strontium-doped biomaterials, such as solid-state reactions and co-precipitation, are extensively utilized due to their straightforwardness. Solid-state reactions typically involve high-temperature calcination (exceeding 1,200 °C) of SrCO_3_ or SrO with metal oxides, often resulting in heterogeneous particles characterized by low surface area and limited control over microstructure (Lv et al., 2015; Naciri et al., 2020). While co-precipitation enhances homogeneity, it may introduce stoichiometric deviations and still necessitates energy-intensive post-processing. Modern techniques, like sol-gel synthesis paired with freeze-drying, allow for accurate integration of strontium into hydroxyapatite (Sr-HA) and bioactive glass frameworks. These techniques produce porous structures that enable prolonged Sr^2+^ release and boost osteogenic differentiation in human bone marrow mesenchymal stem cells (hBMSCs) (Chen Y. Y. et al., 2025; Tsai et al., 2021; D et al., 2021; Ciołek et al., 2023; Naraginti et al., 2015; Manoochehri et al., 2022). Electrodeposition techniques facilitate the formation of strontium-doped calcium phosphate coatings on titanium, thereby enhancing osteoblast adhesion and implant osseointegration (Huang and Yoshimura, 2020; Lafzi et al., 2023; Mumith et al., 2020). However, despite these advancements, the scalability of these methods for clinical application remains challenging due to the complexity of the processes, high energy consumption, and concerns regarding reproducibility.

#### 4.1.2 Surface modification strategies

Surface engineering approaches are considered as effective methods to modulate the bioactivity, mechanical properties, and antibacterial performance of strontium-doped biomaterials. For example, dopamine mediated silk fibroin coating on strontium-doped polyphosphate calcium scaffolds (SCPP/D/SF) showed improved compressive strength, promoted angiogenic factors release and enhanced cellular compatibility (Wang et al., 2016). Chemical grafting of sodium chondroitin sulfate strontium on PEEK surface could further improve osteogenic activity and induce vascularization greatly (Zheng et al., 2022). Co-doping with Sr^2+^ and Silver ions (Ag^+^)has been proved to improve antibacterial performance without compromising bioactivity. Furthermore, coating of multi-layered TiO_2_-Sr^2+^-CaSiO_3_ composites with biopolymers on the surface could improve corrosion resistance and enhance osteoblast adhesion (Cochis et al., 2020; Xie et al., 2018; Raj et al., 2016; Meininger et al., 2016). Despite above-mentioned advances, careful fine-tuning is still needed for the ion release rate, mechanical property, and long-term biocompatibility of the materials. The high release rate of ions or the existence of co-doped elements may induce cytotoxicity or inflammation, so the ions should be strictly controlled in design.

### 4.2 Advance of additive manufacturing and nanotechnology

#### 4.2.1 3D printing

3D printing, one form of additive manufacturing, due to its precise control of scaffold shape and porosity, several materials have been created, like the tailored strontium-doped polycaprolactone/bioactive glass and Sr-HA scaffolds These structures enhance cell adhesion, growth, and alkaline phosphatase activity, highlighting their important use in oral tissue engineering (Fendi et al., 2024; Lino et al., 2019; Yuan et al., 2024; de Souza et al., 2023). The integration of electrospinning techniques results in nanofiber composites that more accurately replicate the extracellular matrix, thereby further enhancing osteogenic differentiation (Dibazar et al., 2023). Furthermore, incorporating bioactive molecules like BMP-2 with strontium-doped scaffolds during co-printing results in a synergistic boost to bone regeneration (Yang et al., 2021; Yadav et al., 2021).

#### 4.2.2 Nanotechnology

Nanotechnology presents novel opportunities for the enhancement of strontium-doped materials. Specifically, strontium-doped hydroxyapatite, strontium titanate nanoparticles, and metal-doped amorphous calcium phosphate (ACPMetals) have shown advancements in bioactivity, antibacterial efficacy, and controlled biodegradability (Moerbeck-Filho et al., 2020; Kaczmarek-Szczepańs et al., 2024; Lin et al., 2016). Bio-inspired designs, drawing from echinoderm skeletal architectures, offer templates for the fabrication of intricate inorganic structures, while plant-mediated strontium nanoparticles exhibit antioxidant properties that are promising for dental applications (Raja Somu et al., 2023; Vallinayaki et al., 2024). Although these methodologies broaden the functional capabilities of strontium-doped materials, they also introduce challenges related to inflammatory responses, cytotoxicity, and reproducibility. This underscores the necessity for rigorous and systematic *in vivo* evaluation.

#### 4.2.3 Integration with stem cells

Dental-derived mesenchymal stem cells, such as DPSCs and SHEDs, are crucial progenitor cells in oral regenerative medicine due to their ability to self-renew and differentiate into multiple cell types. Strontium-doped biomaterials have been shown to synergistically enhance tissue repair mediated by stem cells. For example, Sr@Zn@SiO_2_ nanocomposite implants have been found to enhance BMP-2 expression and increase Smad1/5/9 phosphorylation in BMSCs, thus aiding in osteogenic differentiation and cell migration (Wang S. et al., 2024). Furthermore, low concentrations of strontium (ranging from 0.1 to 2.5 mM) have been found to stimulate the proliferation of DPSCs, enhance alkaline phosphatase activity, promote collagen synthesis, and support matrix mineralization, while also modulating genes related to dentinogenesis, such as DSPP and DMP-1 (Liao et al., 2023). Despite these encouraging outcomes, the therapeutic application of constructs combining strontium and stem cells necessitates meticulous regulation of strontium concentration, scaffold architecture, and cell source. The translation of these findings into reproducible, safe, and clinically viable treatments continues to present a significant challenge.

## 5 Clinical applications and functional roles of strontium-doped biomaterials in oral tissue repair

Oral diseases are highly prevalent and frequently result in significant damage to both hard and soft tissues. Traditional therapeutic approaches, such as the use of antibiotics, primarily focus on reducing inflammation but seldom succeed in restoring the original architecture and functionality of oral tissues (Yang et al., 2025). In comparison, strontium-doped biomaterials have appeared as promising solutions for the regeneration and restoration of tissues. These materials have demonstrated potential applications across various domains, including bone, cartilage, the pulp-dentin complex, periodontal tissues, and immunomodulation, thereby providing a comprehensive strategy to enhance oral function and improve patient quality of life (Table 1).

**TABLE 1:** Application of strontium-doped materials in different types of oral tissue repair.

Tissue types	Materials types	Function	Key results	References
Bone	PCL/strontium, SMPC	Bone formation, mineralization	Increased formation of new bone tissue *in vivo* (Huang et al., 2026)	Huang, K., et al., *3D-printed functionalized strontium-silk fibroin-hydroxyapatite scaffolds facilitate bone regeneration* via *immunomodulatory and sequential angiogenic-osteogenic coupling.* Bioact Mater, 2026. 55: p. 271–289.
Cartilage	Sr-GelMA hydrogel	Cartilage regeneration (Xu et al., 2021); Anti-Inflammatory	β-catenin decreased, Increased thickness of articular cartilage *in vivo*	Xu, L., et al., *Metformin Hydrochloride Encapsulation by Alginate Strontium Hydrogel for Cartilage Regeneration by Reliving Cellular Senescence.* Biomacromolecules, 2021. 22(2): p. 671–680
Pulp-dentin complex	Piezoelectric film (Li et al., 2024b), Sr-BG cement	DPSCs differentiation; Dentinogenesis	Dentin regeneration in animal models	Li, J., et al., *Strontium-Containing Piezoelectric Biofilm Promotes Dentin Tissue Regeneration.* Adv Mater, 2024. 36(21): p. e2313419
Periodontal	Sr-BGN/GNM scaffold, Silk-based nanoplatform (Kanoujia et al., 2025)	Soft tissue regeneration; angiogenesis	Intraoral periodontal regeneration enhancement	Kanoujia, J., et al., *Revealing the promising era of silk-based nanotherapeutics: a ray of hope for chronic wound healing treatment.* Naunyn Schmiedebergs Arch Pharmacol, 2025. 398(6): p. 6617–6641

### 5.1 Hard tissue repair

**FIGURE 6:**
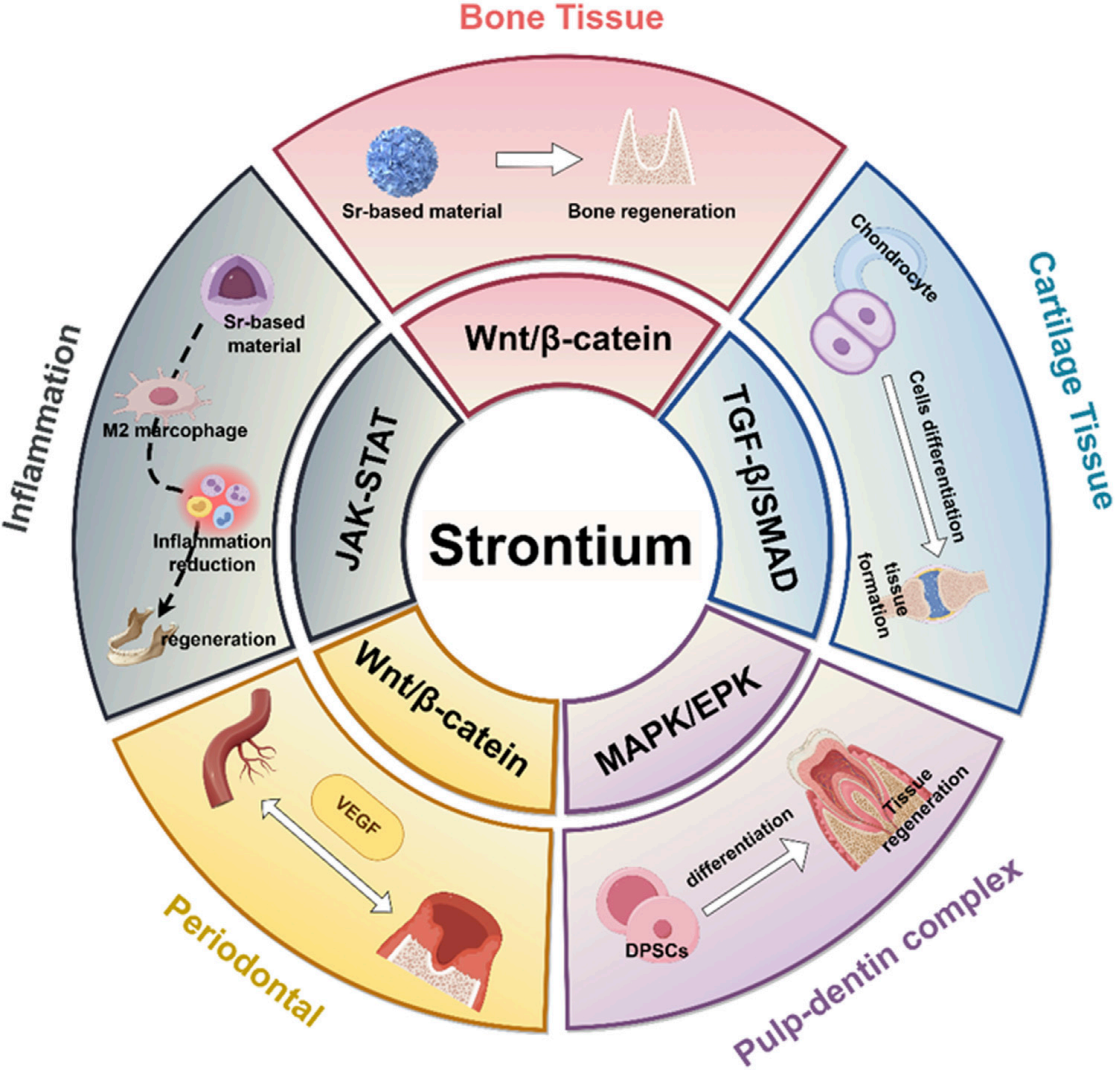
Molecular mechanisms of strontium-doped biomaterials in mediating hard tissue repair. It illustrates that strontium-doped biomaterials promote the regeneration of different tissues by activating distinct signaling pathways.

Biomaterials doping strontium have shown significant efficacy in facilitating bone regeneration, particularly in applications such as alveolar ridge augmentation, jawbone defect repair, and dental implant support (Figure 6). Specifically, polymer nanocomposite scaffolds, such as PCL/strontium C20, which incorporate 20% strontium carbonate, have been discovered to significantly boost the growth and bone-forming differentiation of hBMSCs, leading to about a fourfold rise in mineral deposition. The increased expression of osteogenic markers such as *BMP-2, Osterix,* and *Runx2* highlights the strong pro-osteogenic impact of these materials (Meka et al., 2016; Ji et al., 2024). Moreover, strontium influences osteoclast activity, which decreases bone resorption and enhances overall bone formation. Strontium-modified calcium phosphate cements (SMPCs), which are enhanced with tricalcium silicate, exhibit superior mechanical properties, injectability, and setting time compared to traditional calcium phosphate cements (CPCs). As the silicate content rises, the compressive strength of these cements improves, with a 5% SMPC reaching a compressive strength of 6.00 ± 0.74 MPa. Laboratory studies have indicated enhanced growth and specialization of bone-forming cells, whereas live animal tests have revealed considerable new bone growth without notable inflammation (Xu et al., 2022; Schumacher et al., 2016).

Carolina Bizelli‐Silveira and colleagues (Bizelli-Silveira et al., 2019) have investigated the effects of increased strontium concentrations on the proliferation and osteogenic activity of hBMSCs derived from embryonic mesenchymal (fibula) and ectomesenchymal (mandible) lineages. The findings revealed that BMSCs from both embryonic mesenchymal (fibula) and ectomesenchymal (mandible) origins, when co-cultured *in vitro* with strontium ranelate at a concentration of 360 mg/L, demonstrated enhanced cell proliferation and osteogenic behavior. In addition, the research team led by Lida Kheiri conducted a comparative analysis of the biological responses of subcutaneous connective tissue to a novel bilayer membrane composed of polycaprolactone (PCL) at 60 wt%, silk fibroin (SF) at 20 wt%, and strontium carbonate (SrC) at 20 wt%, against commercially available collagen membranes. The study assessed the type and severity of inflammation, as well as the formation of granulation and fibrous tissue. The findings indicated that the subcutaneous response elicited by the novel membrane was comparable to that of the commercial collagen membrane. Additionally, the novel membrane exhibited promising potential for application in the treatment of oral and maxillofacial bone defects through guided bone regeneration technology (Kheiri et al., 2025).

Furthermore, gels made from strontium-doped hydroxyapatite combined with branched poly(ε-lysine) dendrimers (Sr-HA/G3-KPS) have been demonstrated to reduce inflammatory cytokine expression in macrophages while simultaneously increasing *IL-6* expression, thereby promoting osteogenesis. Significant new bone formation has been observed in osteoporotic rat models following implantation, highlighting their potential use in repairing jawbone defects (Yuan et al., 2018). Collectively, these studies suggest that strontium-doped scaffolds offer both osteoconductive support and immune modulation, thereby optimizing the microenvironment for bone repair.

### 5.2 Cartilage and temporomandibular joint repair

There is increasing research interest in strontium-doped biomaterials due to their potential for cartilage repair and TMJ tissue regeneration. Strontium influences the Wnt/β-catenin signaling pathway, promoting the transformation of bone marrow mesenchymal stromal cells into chondrocytes. At low concentrations, strontium enhances cell proliferation and chondrogenic potential, as evidenced by the upregulation of cartilage-specific gene and protein expression. Hydrogels containing strontium provide superior lubrication and extended scaffold stability, which ensures sustained support during tissue remodeling. The regenerative ability of strontium is further increased by combining it with other bioactive elements such as bioactive glass and chitosan. *In vivo* studies indicate that strontium ranelate silica nanoparticles, when integrated into GelMA scaffolds, significantly enhance cartilage regeneration in femoral condyle defects over a 3-month period, an effect associated with reduced β-catenin activation. Sr^2+^ aid in the healing of TMJ tissues by enhancing the bone-forming activity of bone marrow mesenchymal stem cells and encouraging the polarization of anti-inflammatory M2 macrophages (Rodríguez-Méndez et al., 2018; Cai et al., 2021). Transcriptomic and proteomic analyses further validate that strontium influences gene expression associated with chondrocyte growth, differentiation, metabolism, inflammation, and immune responses (Rajeswari Krishnankutty et al., 2022; Quade et al., 2020). These results underscore the promise of strontium-doped biomaterials in promoting cartilage regeneration by combining structural support, cellular differentiation, and the regulation of inflammatory responses.

### 5.3 Pulp-dentin complex repair

The regeneration of the pulp is crucial for preserving the vitality of the tooth. Poly (vinylidene fluoride-co-trifluoroethylene) [P(VDF-TrFE)] piezoelectric films, enhanced with 2 wt% strontium chloride, create an ideal electrical microenvironment that supports the recruitment and the transformation of DPSCs into cells resembling odontoblasts. The gradual release of Sr^2+^ further facilitates odontoblastic differentiation, as evidenced by *in vivo* studies that have shown dentin formation in large animal models over a period of 3 months (Li J. et al., 2024). Moreover, strontium-doped nano-bioactive glass cements demonstrate rapid self-setting properties, bioactivity, and suitable biodegradability. Compared to controls lacking strontium, these materials promote enhanced cell proliferation and differentiation, and superior dentin formation when used in conjunction with DPSCs (Mandakhbayar et al., 2019). Bio-hybrid scaffolds combining strontium-folic acid with pluripotent DPSCs enable full regeneration of bone defects, showing their potential use in clinical pulp-dentin complex repair (Martin-Del-Campo et al., 2016). Additionally, strontium mitigates dentin hypersensitivity by occluding dentinal tubules and enhancing mechanical properties (Behzadi et al., 2022; Saeki et al., 2016). Strontium-enriched viscous carbohydrate polymers exhibit optimized rheological characteristics and controlled ion release, thereby improving adhesion and therapeutic efficacy (Jones et al., 2017). Furthermore, the antimicrobial properties of strontium-doped cements contribute to dentin remineralization and infection control (Jayasree et al., 2017).

### 5.4 Periodontal tissue regeneration

Periodontitis, a condition marked by chronic inflammation and tissue degradation, poses a significant clinical challenge. Biomaterials doping strontium have been demonstrated to promote osteogenic differentiation in BMSCs and PDLCs, stimulate angiogenesis in endothelial cells, and support the regeneration of periodontal tissues (Lin et al., 2024). Injectable scaffolds that integrate strontium-doped bioactive glass nanoparticles with gelatin nanofiber microspheres effectively mimic the natural extracellular matrix, offering controlled ion release and mechanical stability. Research conducted in living organisms has shown improved bone growth and blood vessel formation in osteoporotic conditions (Wei et al., 2018). Furthermore, multifunctional silk-based nanoplatforms incorporating strontium are capable of scavenging reactive oxygen species, modulating the immune environment, and enhancing both angiogenesis and osteogenesis, thereby expediting periodontal tissue repair (Ming et al., 2025). These findings substantiate the potential of strontium-doped biomaterials to restore both hard and soft periodontal tissues, even in the presence of challenging inflammatory or osteoporotic conditions.

### 5.5 Anti-inflammatory and immunomodulatory effects

Strontium-doped biomaterials demonstrate significant immunomodulatory and anti-inflammatory properties, which are essential for the effective repair of oral tissues. Long-term inflammation impedes regenerative processes, and Sr^2+^ have been demonstrated to affect different immune cells, such as macrophages, neutrophils, T cells, and dendritic cells. Laboratory studies indicate that strontium promotes the shift of macrophages to the anti-inflammatory M2 type, marked by increased *IL-10* and *TGF-β* production and reduced *TNF-α, IL-6* and *IL-1β* expression. This polarization occurs through the inhibition of NF-κB and related pro-inflammatory signaling pathways (Wang et al., 2025; Harshini et al., 2024). Additionally, strontium enhances neutrophil polarization in a manner that supports angiogenesis and tissue healing, while concurrently reducing oxidative stress and proteolytic activity, thereby mitigating early tissue damage (Li et al., 2021).

Strontium-doped materials have been found to improve the attachment and growth of gingival fibroblasts in dental implant applications, therefore aiding in the creation of a soft tissue barrier that prevents microbial invasion and the occurrence of peri-implantitis (Alshammari et al., 2024; Alshammari et al., 2021; Yin et al., 2021). Bioactive glass with strontium substitution (Sr-SBG) has been found to influence macrophages and mesenchymal stem cells, leading to decreased inflammatory infiltration and increased osteogenic differentiation (Martín-Del-Campo et al., 2019; Zhang et al., 2016). In addition to influencing innate immunity, strontium affects adaptive immune responses by reducing Th17 cell activity and boosting regulatory T-cell populations, while encouraging tolerogenic characteristics in dendritic cells. Together, these actions establish an anti-inflammatory environment that supports the regeneration of bone and soft tissue (Martín-Del-Campo et al., 2019; Zhao et al., 2018). This adjustment of the immune response works in harmony with its effects on blood vessel formation; strontium boosts the expression of *VEGF* and other factors that promote angiogenesis, thereby supporting coordinated vascularization and tissue regeneration. Studies conducted *in vivo* using osteoporotic models have confirmed an increase in vascular density alongside improved bone repair, underscoring the significance of the immuno-vascular interactions facilitated by strontium (Denry et al., 2018).

Moreover, strontium attenuates bone resorption during inflammatory processes by modulating the RANKL/OPG signaling pathway, thereby reducing osteoclastogenesis and promoting osteoblast activity. This characteristic is especially pertinent in conditions such as periodontitis and peri-implantitis, where chronic inflammation is associated with bone loss (Zhao et al., 2018). Research (Bizelli-Silveira et al., 2019) has investigated the antibacterial efficacy of strontium -functionalized wafers against various bacteria associated with implant infections, both in mono-species and multi-species biofilms. The study evaluated the bactericidal and bacteriostatic effects of silicon wafers functionalized with a strontium titanium oxide (Sr-Ti-O) coating, as well as those coated solely with titanium (serving as a control), against several bacterial strains cultivated as mono-species or multi-species biofilms. The assessment was conducted using bacterial viability assays and plate counting methods. The findings demonstrated that strontium functionalization imparted both bactericidal and bacteriostatic properties against bacteria related to peri-implantitis.

Overall, these results indicate that biomaterials with strontium not only reduce inflammation but also promote the development of a regenerative immune microenvironment, thereby enhancing the effectiveness of oral tissue repair in various clinical settings.

## 6 Discussion

### 6.1 Long-term safety and efficacy

**FIGURE 7:**
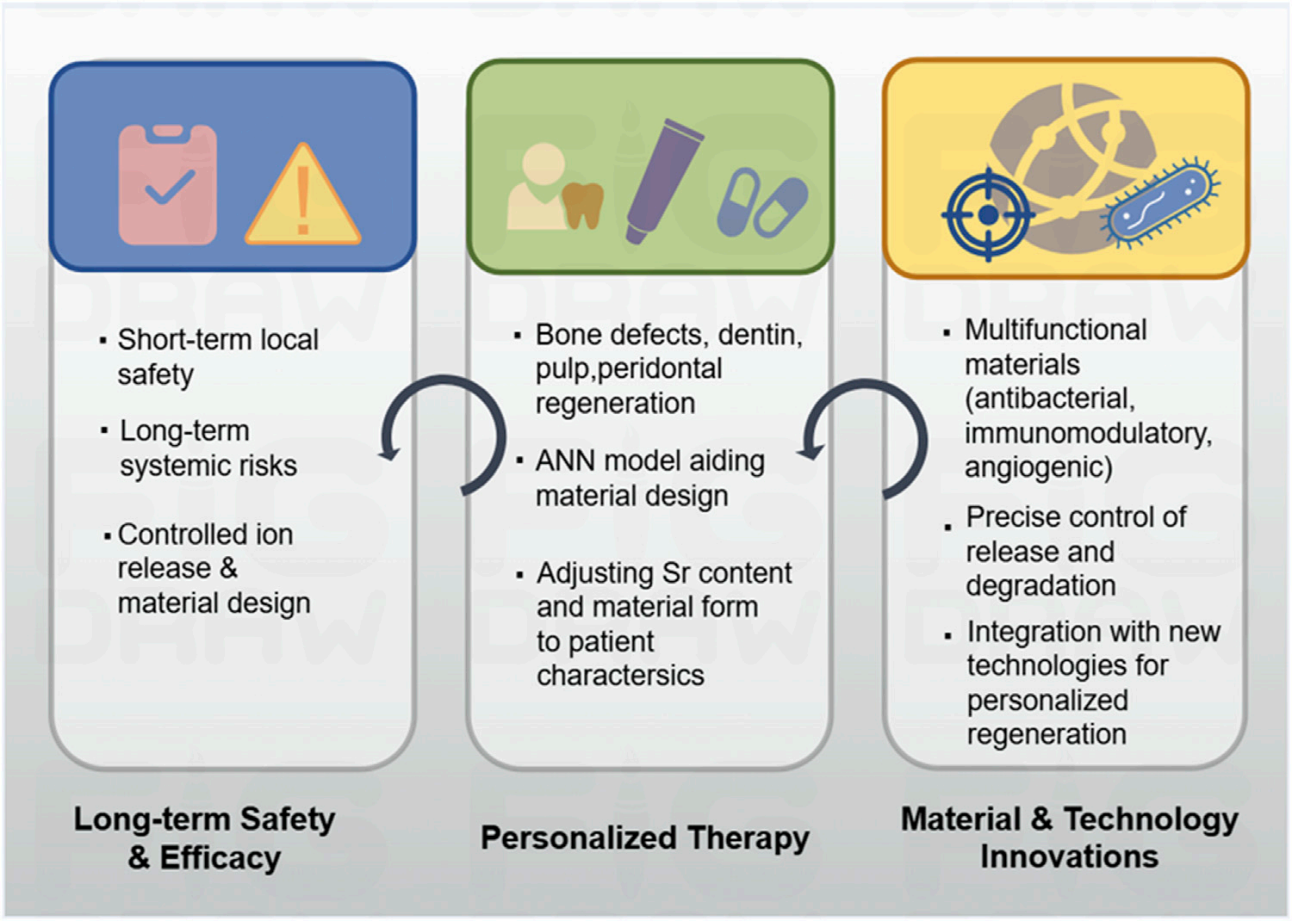
Overview of safety, personalized therapy and future direction of strontium-doped materials.

The successful clinical application of strontium-doped biomaterials is heavily reliant on their long-term safety and sustained efficacy (Figure 7). Extensive preclinical and *in vitro* investigations have demonstrated that these materials can significantly improve oral tissue regeneration. Strontium-doped hydroxyapatite and bioactive glass have been demonstrated their ability of enhance osteoblast growth, differentiation, and mineralization, while speeding up tissue defect repair in animal studies, exhibiting considerable osteoregenerative potential (Vijayalakshmi and Vijayalakshmi, 2020). For biocompatibility evaluations, including zebrafish embryo toxicity assays, indicate that low-crystallinity strontium-containing nanoscale hydroxyapatite microspheres (nSrHA5) slightly increase reactive oxygen species levels without inducing morphological abnormalities or behavioral neurotoxicity, suggesting acceptable biocompatibility under controlled conditions (da Costa Silva et al., 2024). These findings indicate that the meticulous design of crystallinity and particle size is essential for minimizing cytotoxicity while preserving regenerative capabilities.

Notably, Strontium plays a crucial role in bone metabolism, with its osteogenic effects and safety profile demonstrating a clear dose dependency (Abdalla et al., 2024). Specifically, low concentrations of strontium (e.g., 1 mM and 10 mM) significantly enhance the proliferation and osteogenic differentiation of human placental decidua basalis mesenchymal stem cells (PDB-MSCs) and bone marrow mesenchymal stem cells (BM-MSCs). In contrast, higher concentrations (e.g., 0.1 mM) do not exhibit this effect (Huang et al., 2019). This suggests the presence of an optimal dose range for the osteogenic promotion by strontium. In the context of oral tissue engineering, strontium-modified scaffolds, such as those composed of mesoporous bioactive glass/polyvinyl alcohol composites, show increased bioactivity upon the incorporation of strontium (e.g., at 2.5 mol% and 5 mol%). These strontium-modified materials enhance the proliferation and differentiation of osteoblasts while maintaining favorable biocompatibility, underscoring their potential utility in bone defect repair (Jiménez-Holguín et al., 2020). Strontium affects bone formation based on its concentration, with low-to-optimal levels (e.g., 1 mM and 10 mM) generally promoting positive outcomes. However, thorough *in vitro* and *in vivo* testing is necessary to ensure safety and effectiveness, especially to avoid risks from high doses (Jiménez-Holguín et al., 2020; Chen et al., 2024).

Despite these promising outcomes, concerns regarding long-term safety persist. The systemic administration of strontium salts, such as strontium ranelate for osteoporosis treatment, has been linked to cardiovascular risks, underscoring the necessity of monitoring potential systemic effects (Borciani et al., 2022). *In vitro* studies further demonstrate that elevated concentrations of Sr^2+^ may inhibit cellular activity, highlighting the importance of optimizing ion release and local microenvironmental conditions. Current short-term dental studies affirm the safety of strontium-doped biomaterials for localized applications; however, comprehensive longitudinal clinical trials are imperative to evaluate their effects on both oral and systemic tissues. Collectively, these results suggest that although strontium-doped biomaterials hold promise, their long-term safety and appropriate dosage thresholds require careful assessment in clinical settings.

### 6.2 Perspectives

#### 6.2.1 Personalized therapy

Strontium-doped materials hold great potential for generating individualized therapies for regenerative medicine in oral tissues (Figure 7). Given the variation in oral disease phenotypes, oral diseases present different degrees of tissue damage and oral individual health conditions, and thus, individualized therapies are essential. In repairing mandibular defects, scaffolds can be designed with patient-appropriate strontium concentrations and scaffold properties to match the defect geometry, bone quality, and regenerative demand, and such kind of individualized customization would benefit the osteoconduction, mineralization process, and mechanical support. For dentin repair, metal ions such as strontium, Mg, and Zn have been reported to regulate dentin hardness, tubule density, and fracture resistance. The use of ANN enables the evaluation of the effects of metal ions on dentin properties, and an accurate choice of compositions of materials can be made according to the dental conditions of each patient (Saghiri et al., 2023).

Strontium-doped toothpaste is an example of individualized preventive care. The findings from a clinical trial on the use of strontium-doped toothpaste in children provided evidence for the benefits of customized preventive care using strontium. The use of fluoride-based HA particles (Fluoride HA-Filtered, F-HAF) toothpaste in place of regular fluoride toothpaste resulted in a substantial decrease in cavity formation in children between the ages of 6 and 12 years old after a 24-month trial involving 610 children (Cagetti et al., 2022). Synthetic strontium carbonate and CaCO3 nanoparticles can occlude dentinal tubules and promote mineralization by dental pulp stem cells, and these non-toxic dental pulp stem cell-derived strontium-doped nanomaterials for dentin hypersensitivity provide patient-specific solutions (Dotta et al., 2022). For patients with osteoporosis who require jawbone regeneration, scaffolds doped with strontium can take advantage of its dual roles to promote bone formation while simultaneously inhibiting resorption, and thus, meet the unique regenerative requirements of the patients effectively. For pulp regeneration, the combination of dental pulp stem cells derived from patients and appropriately designed strontium-doped scaffolds enables controlled differentiation and tissue formation, and this is a synergistic approach to personalized pulp regeneration.

#### 6.2.2 Materials and technology innovations

Future advancements in strontium-doped biomaterials are anticipated to concentrate on augmenting bioactivity, multifunctionality, and clinical applicability.

Emerging strategies are focused on the integration of strontium-doped materials with advanced fabrication technologies. The surface modification of implants using strontium-doped titanium and nanoscale disordered topographies has demonstrated potential in enhancing osteoinduction while inhibiting osteoclast activity, thereby facilitating accelerated osseointegration (Young et al., 2024). Techniques such as 3D printing enable precise customization of scaffold geometry and composition to accommodate patient-specific defects, while gene editing of stem cells can enhance their regenerative synergy with strontium scaffolds. Moreover, the advancement of intelligent and responsive biomaterials that can release Sr^2+^ in reaction to local pH fluctuations, enzymatic activity, or inflammatory signals holds the potential to significantly enhance therapeutic precision.

In additional, a promising avenue for future research is the exploration of the interactions between strontium-doped biomaterials and oral microbiota. The ability to maintain microbial equilibrium while facilitating tissue regeneration has the potential to mitigate infection risks and enhance treatment efficacy. The advancement of multifunctional coatings for dental implants, incorporating antibacterial, anti-inflammatory, angiogenic, and osteogenic properties, represents a potential breakthrough in the evolution of next-generation oral regenerative materials.

## 7 Conclusion

Strontium and its derivative biomaterials, renowned for their distinctive biological properties, have emerged as crucial instruments in the field of oral bone regeneration. Despite their potential, several challenges persist, including inconsistent degradation profiles that may lead to abrupt strontium ion release, potential cytotoxicity due to prolonged accumulation, and barriers to clinical application. Through the promotion of interdisciplinary integration—encompassing materials science, tissue engineering, and regenerative biology—strontium-doped materials hold the promise of transcending their role as mere space-fillers to actively influencing cellular and molecular processes. Such advancements could facilitate the development of more precise, personalized, and effective strategies for oral tissue regeneration in the future.
